# Supplementary material for: Dynamics in Fip1 regulate eukaryotic mRNA 3′ end processing
Source: Genes Dev. 2021 Nov 1;35(21-22):1510–26. doi: 10.1101/gad.348671.121 (PMC8559680; doi:10.1101/gad.348671.121)
Supplement: Supplemental Material [file supp_35_21-22_1510__DC1.html]

Dynamics in Fip1 regulate eukaryotic mRNA 3′ end processing — Supplemental Material 

# Dynamics in Fip1 regulate eukaryotic mRNA 3′ end processing

## Supplemental Material

- Supplemental\_Figures.pdf
- Supplemental\_Table\_S1.pdf
- Supplemental\_Table\_S2.pdf
