# Supplementary material for: Dynamics in Fip1 regulate eukaryotic mRNA 3′ end processing
Source: Genes Dev. 2021 Nov 1;35(21-22):1510–26. doi: 10.1101/gad.348671.121 (PMC8559680; doi:10.1101/gad.348671.121)
Supplement: Supplemental Material [file supp_gad.348671.121_Supplemental_Figures.pdf]

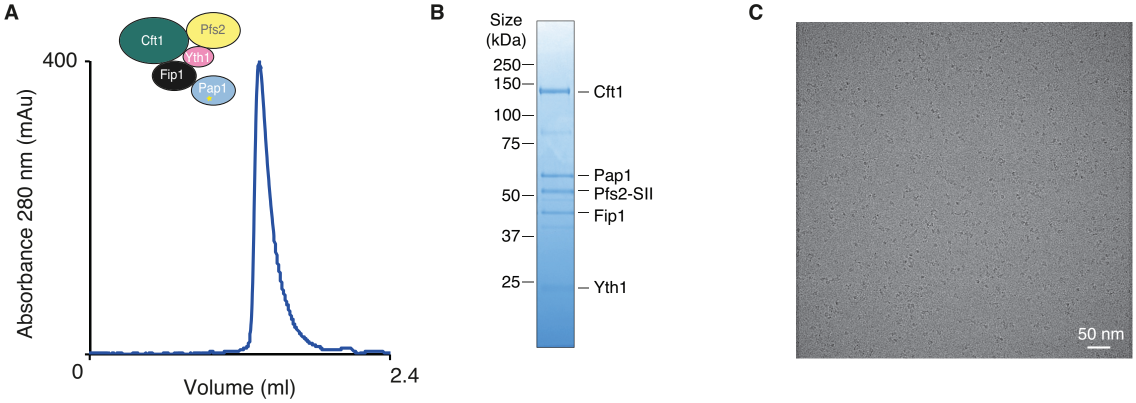

Supplemental Fig. S1. CryoEM of yeast polymerase module. (A) Size exclusion chromatogram of purified polymerase module. **(B)** SDS-PAGE showing the five subunits of purified polymerase module. Pfs2 has a StrepII tag (SII). **(C)** Cryo-EM micrograph of five-subunit polymerase module.

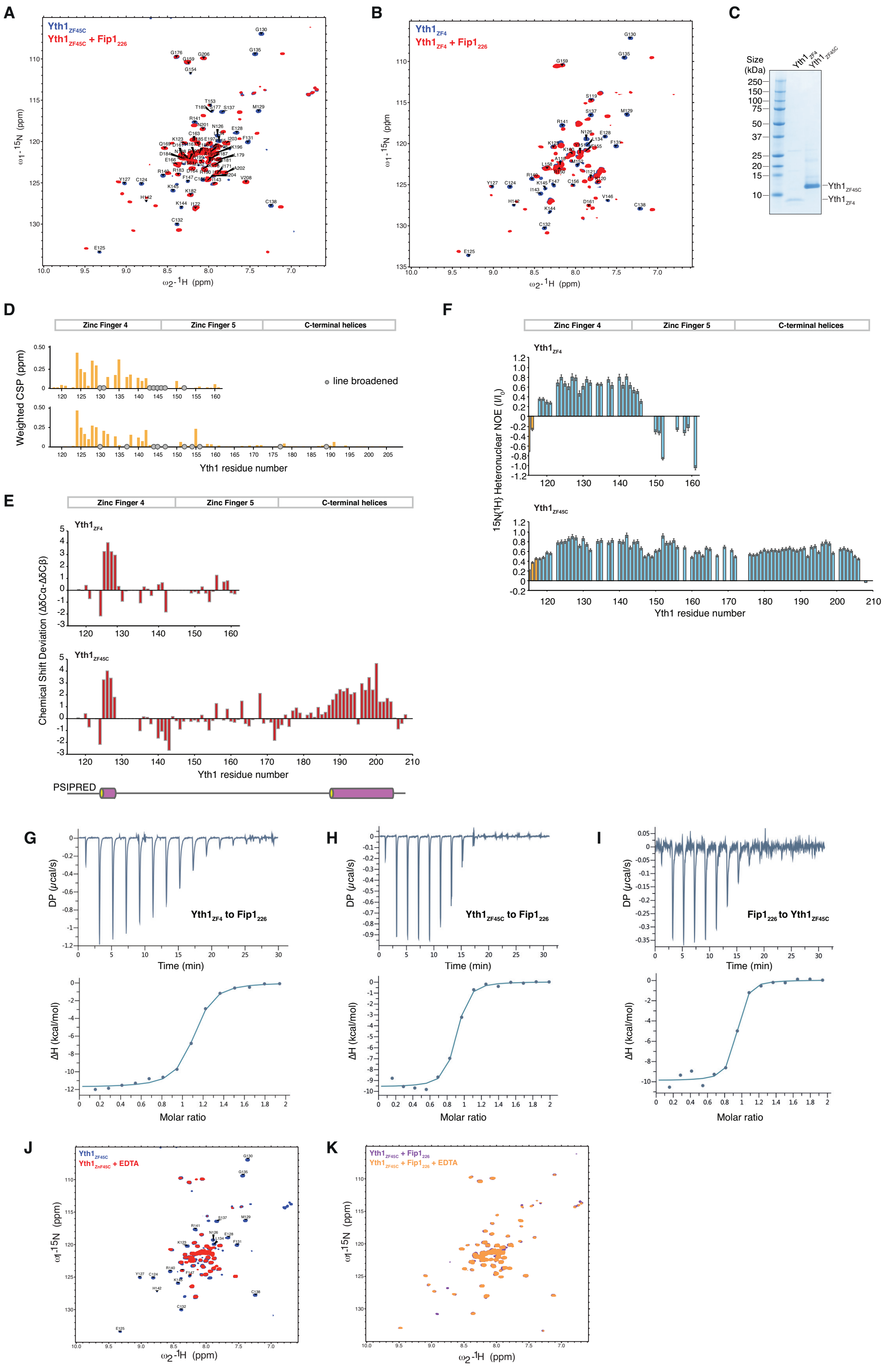

A
Yth1ZF45C
Yth1ZF45C + Fip1226
ω2-1H (ppm)
ω1-15N (ppm)
B
Yth1ZF4
Yth1ZF4 + Fip1226
C
Size (kDa)
Yth1ZF4
Yth1ZF45C
D
Zinc Finger 4
Zinc Finger 5
C-terminal helices
Weighted CSP (ppm)
line broadened
Yth1 residue number
E
Chemical Shift Deviation (ΔδCα-ΔδCβ)
PSIPRED
F
15N{1H} Heteronuclear NOE (I/I0)
G
DP (μcal/s)
Yth1ZF4 to Fip1226
Time (min)
ΔH (kcal/mol)
Molar ratio
H
Yth1ZF45C to Fip1226
I
Fip1226 to Yth1ZF45C
J
Yth1ZF45C
Yth1ZnF45C + EDTA
K
Yth1ZF45C + Fip1226
Yth1ZF45C + Fip1226 + EDTA

Supplemental Fig. S2. Fip1 interacts with zinc finger domain 4 of Yth1. $^{1}H,^{15}N$ 2D HSQCs of **(A)** Yth1$_{ZF45C}$ and **(B)** Yth1$_{ZF4}$ reveal major chemical shift perturbations upon binding to Fip1$_{226}$. Backbone assignment of the two Yth1 constructs are labelled on the spectra. Yth1$_{ZF45C}$ / Yth1$_{ZF4}$ and Fip1$_{226}$ were mixed in equimolar amounts to a final concentration of 75 µM. **(C)** SDS-PAGE showing the two Yth1 constructs used for NMR analysis. **(D)** Chemical shift perturbations in the HSQC spectra mapped onto the Yth1 sequence. A similar pattern was observed in both constructs, and most of the perturbations are localized in zinc finger 4. Grey circles indicate resonances that showed exchange broadening upon Fip1 binding. **(E)** Cα/Cβ chemical shift deviations reveal secondary structure propensities. Positive values suggest helical propensity and negative values suggest propensity for β-strand formation. PSIPRED prediction for Yth1$_{ZF45C}$ is shown in the schematic below (tube for alpha helix). **(F)** $^{15}N\{^{1}H\}$ heteronuclear NOE values were recorded at 278K and plotted as I/I_0. Extra residues at the N-termini of the Yth1 constructs that result from the cloning strategy are colored in orange. Comparison between Yth1$_{ZF4}$ and Yth1$_{ZF45C}$ shows zinc finger 4 retains the properties of Yth1$_{ZF45C}$ with a possible domain boundary at residue 148. The hetNOE values were obtained by averaging two experiments. The reported error values are calculated standard deviations. **(G-I)** Binding affinities of Yth1 and Fip1 were determined by isothermal calorimetry (ITC). **(G)** 300 µM Yth1$_{ZF4}$ was injected into 30 µM Fip1$_{226}$ and the measured K_D was 240 ± 40 nM. The number of binding sites on Fip1$_{226}$ for Yth1$_{ZF4}$ was determined to be 1.05 ± 0.01. **(H)** 300 µM Yth1$_{ZF45C}$ was injected into 30 µM Fip1$_{226}$ and the measured K_D was 150 ± 40 nM. The number of binding sites on Fip1$_{226}$ for Yth1$_{ZF4}$ was determined to be 0.85 ± 0.01. **(I)** 150 µM Fip1$_{226}$ was injected into 15 µM Yth1$_{ZF45C}$ and the measured K_D was 50 ± 20 nM. The number of binding sites on Yth1$_{ZF4}$ for Fip1$_{226}$ was determined to be 0.88 ± 0.01 in these conditions. This measurement was limited by the concentration of Fip1$_{226}$. **(J)** $^{1}H,^{15}N$ 2D HSQC of Yth1$_{ZF45C}$ with (red) and without (blue) 1 mM EDTA. Crosspeaks from residues 118–142 of Yth1$_{ZF4}$ are labelled in the spectra. EDTA addition results in a spectrum with narrow dispersion in proton chemical shift, suggesting the protein has lost its globular fold. **(K)** $^{1}H,^{15}N$ 2D HSQC of Yth1$_{ZF45C}$-Fip1$_{226}$ with (orange) and without (purple) 1 mM EDTA. The complex was prepared by mixing equimolar amounts of Yth1$_{ZF45C}$ and Fip1$_{226}$ at 75 µM, and it remains stable in 1 mM EDTA. BEST $^{1}H,^{15}N$-TROSY spectra were acquired with 16 scans and a recycle delay of 400 ms, with a final spectral resolution of 0.9 Hz per points and an experimental time of 18 minutes.

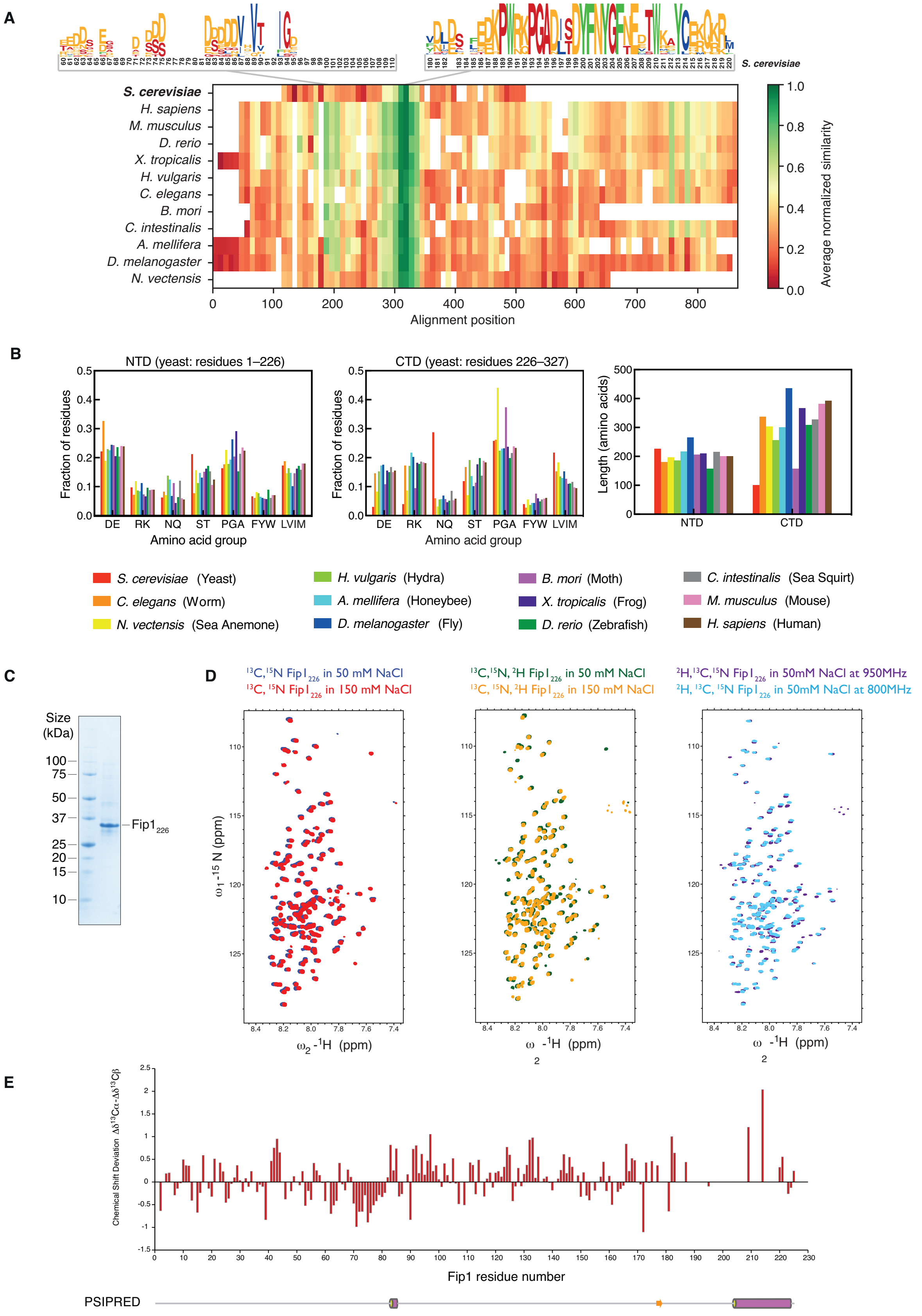
A
S. cerevisiae
H. sapiens
M. musculus
D. rerio
X. tropicalis
H. vulgaris
C. elegans
B. mori
C. intestinalis
A. mellifera
D. melanogaster
N. vectensis
Alignment position
Average normalized similarity
B
NTD (yeast: residues 1–226)
CTD (yeast: residues 226–327)
Fraction of residues
Amino acid group
DE RK NQ ST PGA FYW LVIM
Length (amino acids)
NTD
CTD
S. cerevisiae (Yeast)
C. elegans (Worm)
N. vectensis (Sea Anemone)
H. vulgaris (Hydra)
A. mellifera (Honeybee)
D. melanogaster (Fly)
B. mori (Moth)
X. tropicalis (Frog)
D. rerio (Zebrafish)
C. intestinalis (Sea Squirt)
M. musculus (Mouse)
H. sapiens (Human)
C
Size (kDa)
100 75 50 37 25 20 15 10
Fip1226
D
13C,15N Fip1226 in 50 mM NaCl
13C,15N Fip1226 in 150 mM NaCl
13C,15N,2H Fip1226 in 50 mM NaCl
13C,15N,2H Fip1226 in 150 mM NaCl
2H,13C,15N Fip1226 in 50mM NaCl at 950MHz
2H,13C,15N Fip1226 in 50mM NaCl at 800MHz
ω1 -15N (ppm)
ω2 -1H (ppm)
E
Chemical Shift Deviation Δδ13Cα-Δδ13Cβ
Fip1 residue number
PSIPRED

Supplemental Fig. S3. Sequence and structure analysis of Fip1. (A) Plot of sequence homology of Fip1 across various eukaryotic species. Sequences of homologs of yeast Fip1 were collected from the UniProt database and aligned using ClustalOmega with default parameters. Residues 60–110 and 190–220 were further analyzed using sequence logos. High conservation is mainly observed between residues 190–210. **(B)** Comparison of amino acid composition and lengths of the N-terminal domains (NTD, residues 1–226 in yeast) and C-terminal domains (CTD, residues 227–327 in yeast) of Fip1 across species. **(C)** SDS-PAGE showing the purified $Fip1_{226}$ construct used for NMR analysis. **(D)** To retrieve some of the line-broadened signals, $Fip1_{226}$ spectra were compared after collection in different conditions including 50 mM and 150 mM NaCl, with and without ^{2}H-labelling, and at 800 MHz and 950 MHz. Assignment of backbone resonances was performed using data collected at 800 and 950 MHz with $^{13}C,^{15}N,^{2}H$ $Fip1_{226}$ in 50 mM NaCl (see Methods). Line-broadening is particularly prevalent for peaks from residues 170–220. BEST $^{1}H,^{15}N$-TROSY spectra were acquired with 4 scans and a recycle delay of 400 ms, giving a final spectral resolution of 0.9 Hz per points in the indirect dimension and an experimental time of 20 minutes. **(E)** Plot of Cα/Cβ chemical shift deviations. Temperature coefficients (Kjaergaard et al. 2011) and correction factors for perdeuteration (Maltsev et al. 2012) were applied to the random coil chemical shifts. Positive values suggest helical propensity and negative values suggest β-strand propensity. PSIPRED prediction of secondary structure for $Fip1_{226}$ is shown in cartoon, below the plot.

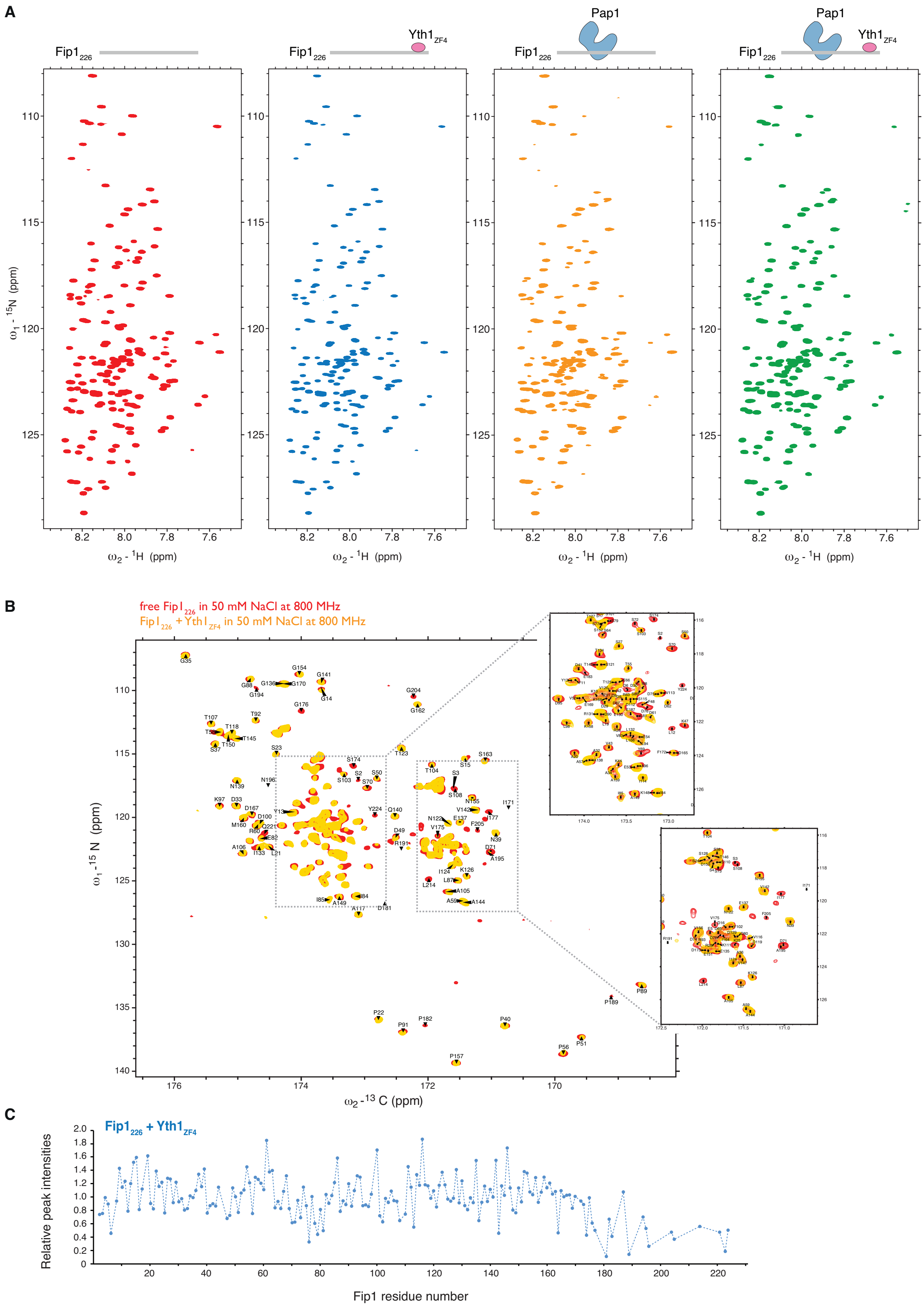

A
Fip1226
Yth1ZF4
Pap1
ω1 - 15N (ppm)
ω2 - 1H (ppm)
B
free Fip1226 in 50 mM NaCl at 800 MHz
Fip1226 + Yth1ZF4 in 50 mM NaCl at 800 MHz
ω1 - 15 N (ppm)
ω2 - 13 C (ppm)
C
Fip1226 + Yth1ZF4
Relative peak intensities
Fip1 residue number

Supplemental Fig. S4. Fip1$_{226}$ binding studies. (A) ^{1}H,^{15}N 2D HSQC for free Fip1$_{226}$ (red), and Fip1$_{226}$ in the presence of Yth1$_{ZF4}$ (blue), Pap1 (orange), and both Yth1$_{ZF4}$ and Pap1 (green). Cartoons depicting the proteins included in each experiment are shown above. Protein concentrations of 60 μM were used in each sample, with equimolar binding partners. BEST ^{1}H,^{15}N-TROSY spectra were acquired at 800 MHz with ^{13}C,^{15}N Fip1$_{226}$ in 150 mM NaCl buffer, with 4 scans and a recycle delay of 400 ms, with a final spectral resolution of 0.9 Hz per points in the indirect dimension and an experimental time of 20 minutes. **(B)** ^{13}C-detect ^{13}C,^{15}N 2D CON spectra for 170 μM free Fip1$_{226}$ (red) and Fip1$_{226}$ in the presence of equimolar Yth1$_{ZF4}$ (yellow). To retrieve some of the missing peaks due to exchange broadening, CON spectra were collected at 800 MHz with ^{13}C,^{15}N,^{2}H Fip1$_{226}$ in 50 mM NaCl buffer. Spectra were collected with 160 scans and a recycle delay of 1 s, with a final resolution of 4.0 Hz per points in the indirect dimension and an experimental time of 5 hours and 20 minutes. **(C)** Changes in relative peak intensities for Fip1$_{226}$ in the presence of Yth1$_{ZF4}$, compared to free Fip1$_{226}$.

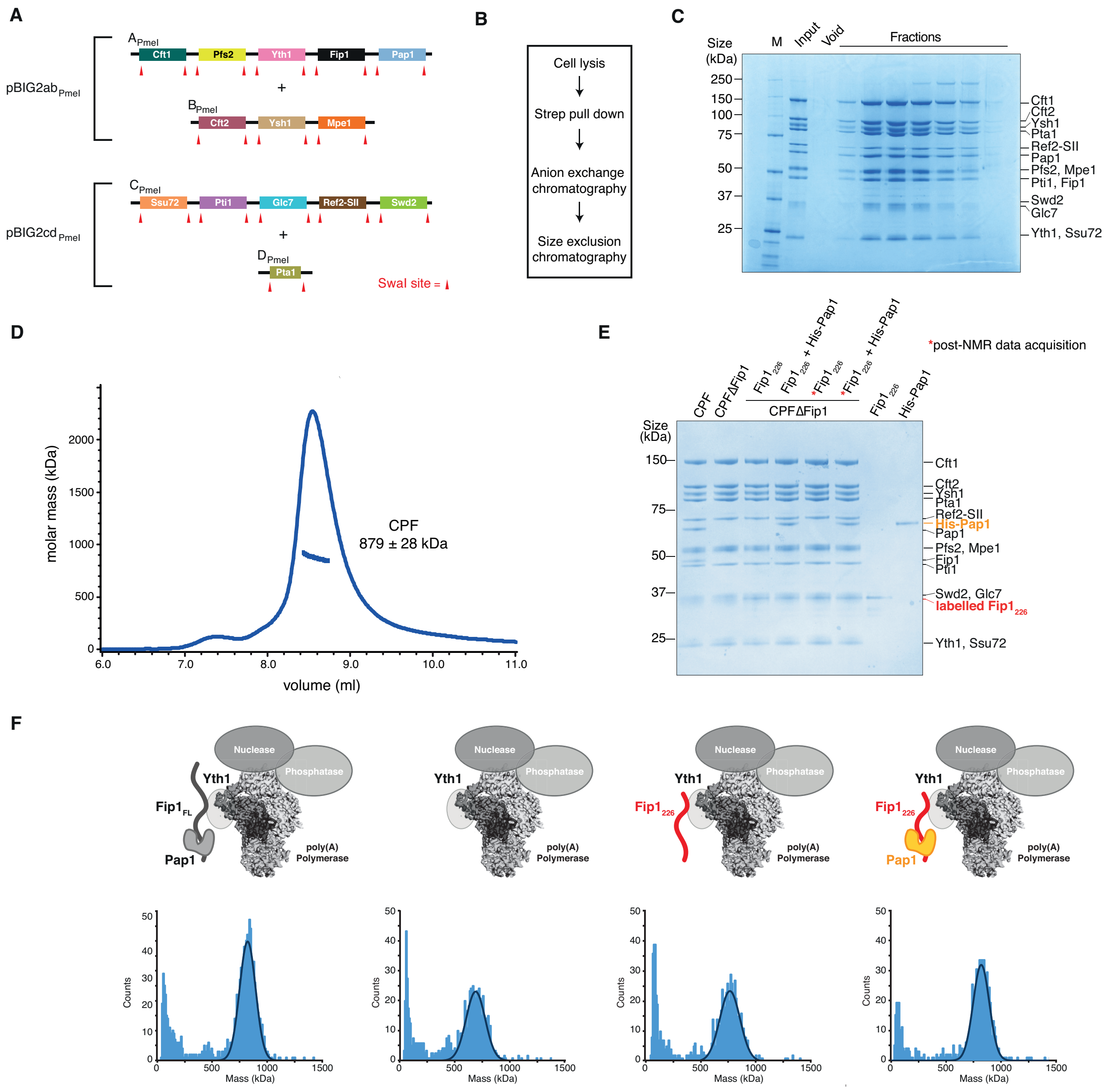

Sample	CPF	CPFΔFip1	CPFΔFip1 + Fip1 226	CPFΔFip1 + Fip1 226 + Pap1
Theoretical MW	860 kDa	760 kDa	785 kDa	851 kDa
Event counts	755 (70%)	654 (64%)	565 (66%)	604 (75%)
Average MW	856 ± 12 kDa	749 ± 12 kDa	779 ± 9 kDa	844 ± 13 kDa

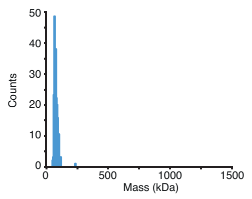

Sample **Buffer**

Supplemental Fig. S5. Purification of recombinant CPF and reconstitution of CPF-Fip1$_{226}$ for NMR analysis. (A) Multi-bacmid strategy for producing recombinant CPF using a baculovirus expression system. Cartoons of PmeI-digested fragments are shown. The pBIG2ab bacmid contains subunits of the nuclease and polymerase modules, and the pBIG2cd bacmid contains subunits of the phosphatase module. The two bacmids were used for coinfection of *Sf*9 insect cells. **(B)** Workflow for recombinant CPF purification. **(C)** SDS-PAGE showing fractions from size exclusion chromatography of recombinant CPF. M, molecular weight marker. **(D)** SEC-MALS analysis of recombinant CPF. The dominant species has an average molecular weight of 879 ± 28 kDa, which is consistent with the expected molecular weight of 860 kDa. (**E)** Pulldown assays of reconstituted CPF complexes using StrepII-tagged (SII) Pfs2. Both Fip1 and Pap1 are absent in the CPFΔFip1 sample. *E. coli* expressed His-Pap1 and Fip1$_{226}$ are shown on the right. The same samples were analyzed by pull-downs and visualized by SDS-PAGE after NMR data acquisition to confirm sample stability (red asterisks). **(F)** Recombinant CPF complexes were analyzed by mass photometry. Schematic diagrams indicate the sample used in each analysis. The masses obtained for the events observed are plotted as histograms and the peaks are fitted to simple gaussian distributions indicated by the black lines. The number of events within these peaks and the percentage of the total events detected are indicated. Measurements were done in triplicate and the reported error values are the calculated standard deviations. Given the gaussian distributions of masses, we cannot exclude that CPF contains a mixture of complexes with 0, 1 and 2 copies of Fip1 and Pap1. A buffer only blank indicates that it contributes the majority of background events detected with masses below 75 kDa in the CPF complex measurements.

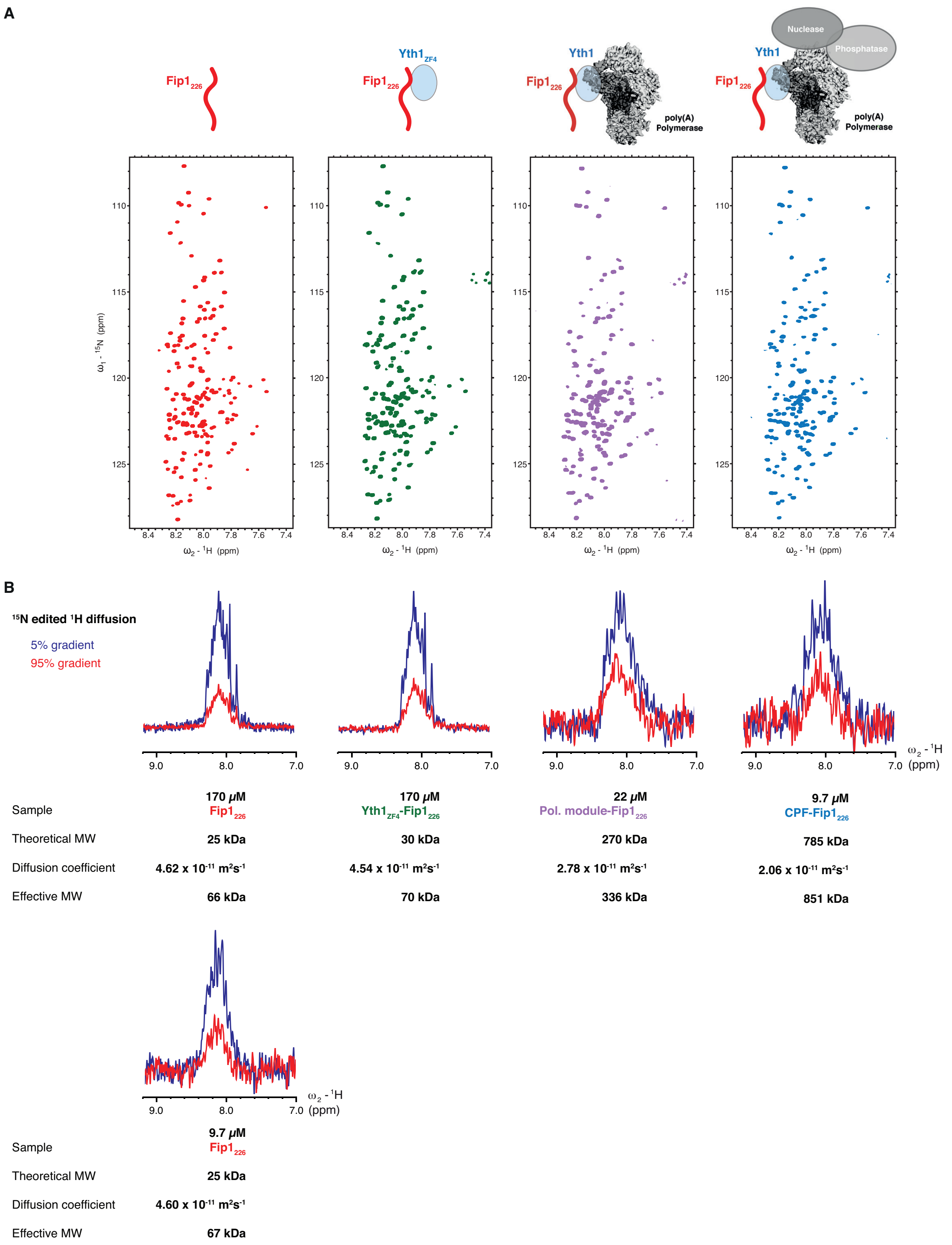

Supplemental Fig. S6. Diffusion experiments to monitor sample stability and size during NMR data acquisition. (A) $^{1}H,^{15}N$ 2D HSQC of free $Fip1_{226}$ (red), $Yth1_{ZF4}$-$Fip1_{226}$ (green), polymerase module-$Fip1_{226}$ (purple) and CPF-$Fip1_{226}$ (blue). Schematic diagrams indicate the protein components in each sample, with equimolar binding partners. For $Fip1_{226}$ and $Yth1_{ZF4}$-$Fip1_{226}$, BEST $^{1}H,^{15}N$-TROSY spectra

were acquired at 800 MHz with 4 scans and a recycle delay of 400 ms, giving a final spectral resolution of 2.2 Hz per points in the indirect dimension and an experimental time of 8 minutes. For polymerase module-Fip1$_{226}$ and CPF-Fip1$_{226}$, BEST ^{1}H,^{15}N-TROSY spectra were acquired at 950 MHz with 64 scans and a recycle delay of 400 ms, giving a final spectral resolution of 2.2 Hz per points in the indirect dimension and an experimental time of 142 minutes. **(B)** ^{15}N-edited ^{1}H diffusion experiments were used to estimate the diffusion coefficients of the ^{15}N-labelled species in each sample. Smaller proteins diffuse more quickly and have a larger signal attenuation. By measuring the signal attenuation in two gradient fields, the diffusion coefficients were determined and converted to their effective molecular masses. Measurements at two different concentrations of Fip1$_{226}$ (170 and 9.7 μM) show that determination of the diffusion coefficient is reproducible and that the method is sensitive even at low concentrations.

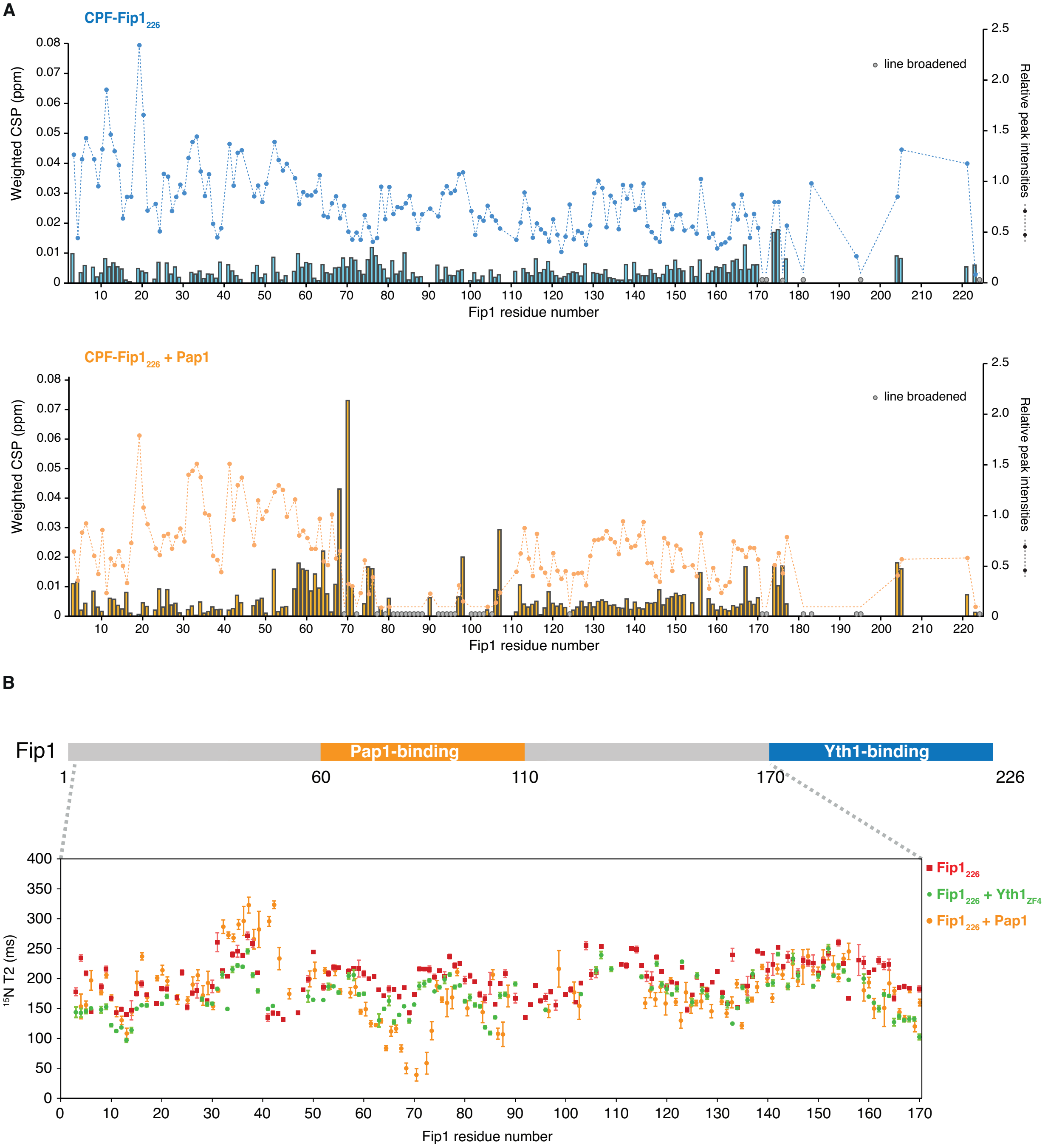

Supplemental Fig. S7. Chemical shift perturbations and exchange-broadening in CPF-Fip1$_{226}$ binding studies. (A) Detailed analysis of the HSQC spectra shown in Fig. 5A. Chemical shift perturbations (CSPs) are shown as histograms. Relative peak intensities compared to free Fip1$_{226}$ at the same concentration (11 µM) are indicated with dotted lines. Peaks that were line-broadened beyond detection are indicated with grey circles. **(B)** Comparison of T2 relaxation times of free Fip1$_{226}$ (red), Fip1$_{226}$ + Yth1$_{ZF4}$ (green) and Fip1$_{226}$ + Pap1 (orange). Since residues in the Yth1-binding site are exchange broadened, only the first 170 residues on Fip1 were analyzed. Peaks that are significantly line broadened in Fip1$_{226}$ + Pap1 were not included for analysis.

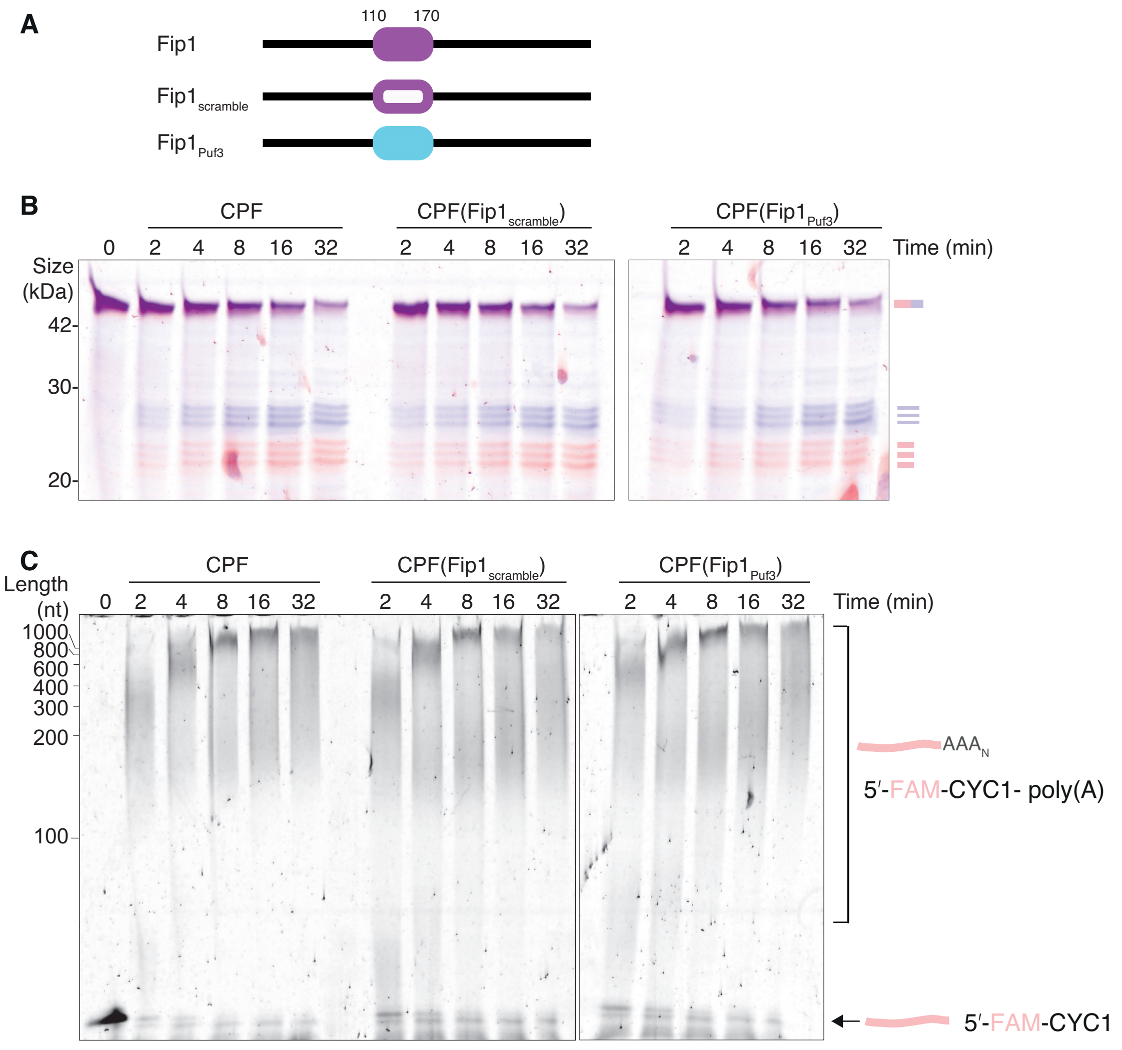

Supplemental Fig. S8. Cleavage and polyadenylation activity of CPF containing Fip1 variants. **(A)** Schematic diagrams showing Fip1 variants where residues 110–170 of the central LCR were replaced with either a scrambled central LCR sequence ($Fip1_{scramble}$) or with residues 3–63 of fission yeast Puf3 ($Fip1_{Puf3}$). **(B)** Denaturing gel electrophoresis of *in vitro* cleavage assays of a *CYC1* 3'-UTR containing a 5′-FAM and a 3′-A647 label. **(C)** *In vitro* polyadenylation of and RNA that contains a 5′-FAM label. In (B) and (C), CPF with wild-type Fip1, $Fip1_{scramble}$ or $Fip1_{Puf3}$ were assayed. These gels are representative of experiments performed two times.
