# Supplementary material for: Dynamics in Fip1 regulate eukaryotic mRNA 3′ end processing
Source: Genes Dev. 2021 Nov 1;35(21-22):1510–26. doi: 10.1101/gad.348671.121 (PMC8559680; doi:10.1101/gad.348671.121)
Supplement: Supplemental Material [file supp_gad.348671.121_Supplemental_Table_S1_.pdf]

Supplemental Table S1: DNA oligonucleotides

Gene	Oligo	Sequence (5′-3′)
***Sf*9 expression vectors**		
Yth1$_{\Delta ZF45C}$	Yth1-45CF	GGAATTCGGATCCCTCGAGATGAGCCTGATTCATCC
Yth1$_{\Delta ZF45C}$	Yth1-45CR	GCCCCATCTAGAGGTACCTCATTACGGATCAATATGCAGATATTG
Yth1$_{\Delta ZF5C}$	Yth1-5CF	GGAATTCGGATCCCTCGAGATGAGCCTGATTCATCC
Yth1$_{\Delta ZF5C}$	Yth1-5CR	GCCCCATCTAGAGGTACCTCATTAATCTTTACCCAGAGGGC
Yth1$_{\Delta ZF4}$	Yth1-del4F1	GGAATTCGGATCCCTCGAGATGAGCCTGATTCATCC
Yth1$_{\Delta ZF4}$	Yth1-del4R1	ATGTTCCATATCACATTCCGGATCAATATGCAGATATT
Yth1$_{\Delta ZF4}$	Yth1-del4F2	AATATCTGCATATTGATCCGGAATGTGATATGGAACAT
Yth1$_{\Delta ZF4}$	Yth1-del4R2	GCCCCATCTAGAGGTACCTCATTATTAAACTTCACCGTTAATAAT
Fip1$_{\Delta 1\text{-}60}$	Fip1-d60F	GGAATTCGGATCCCTCGAGATGGATGATCGTAGTGATGAAGA
Fip1$_{\Delta 1\text{-}60}$	Fip1-d60R	GCCCCATCTAGAGGTACCTCATTATTATTTGCTATTCTGGTTCTGATTCTG
Fip1$_{\Delta 110\text{-}180}$	Fip1-dLCR1	GGAATTCGGATCCCTCGAGATGAGCAGCAGCGAAGATG
Fip1$_{\Delta 110\text{-}180}$	Fip1-dLCR2	CGGTTTTTCTTTCAGAACTTCCGGATCGCTGCTGGTTGCTGCGGTGCTA
Fip1$_{\Delta 110\text{-}180}$	Fip1-dLCR3	TAGCACCGCAGCAACCAGCAGCGATCCGGAAGTTCTGAAAGAAAAACCG
Fip1$_{\Delta 110\text{-}180}$	Fip1-dLCR4	GCCCCATCTAGAGGTACCTCATTATTATTTGCTATTCTGGTTCTGATTCTG
Fip1$_{226}$	Fip1-226F	GGAATTCGGATCCCTCGAGATGAGCAGCAGCGAAGATGAA
Fip1$_{226}$	Fip1-226R	GCCCCATCTAGAGGTACCTCATTAATTGTAATCCTGCTGCAG
***E. coli* expression vectors**		
Fip1$_{226}$	Fip1-226EcF	GGAATTCCATATGAGCAGCAGCGAAGATGAA
Fip1$_{226}$	Fip1-226EcR	CCCAAAGCTTTTATTAATTGTAATCCTGCTGCAG
Yth1$_{ZF45C}$	Yth1-45CEcF	ATTAAGGATCCATGGCAAGCAAAATTCCGAAA
Yth1$_{ZF45C}$	Yth1-45CEcR	ATAATGAATTCTTATTATTAAACTTCACCGTTAATAATGGCG
Yth1$_{ZF4}$	Yth1-ZnF4F	ATTAAGGATCCCCGGATTGTCAATAT
Yth1$_{ZF4}$	Yth1-ZnF4R	ATAATGAATTCTTATTAATCTTTACCCAGAGGG
