# Supplementary material for: Dynamics in Fip1 regulate eukaryotic mRNA 3′ end processing
Source: Genes Dev. 2021 Nov 1;35(21-22):1510–26. doi: 10.1101/gad.348671.121 (PMC8559680; doi:10.1101/gad.348671.121)
Supplement: Supplemental Material [file supp_gad.348671.121_Supplemental_Table_S2_.pdf]

Supplemental Table S2: Buffers

Name	Composition
CPF lysis buffer	200 mM HEPES pH 8, 200 mM KCl, 0.5 mM $Mg(OAc)_2$, 1 mM TCEP, 10% w/v glycerol, 2 μg/ml DNaseI (add fresh), 2 μg/ml RNaseA (add fresh) and protease inhibitor tables (14 tablets per 120 ml of buffer) (Roche)
CPF wash buffer	50 mM HEPES pH 8, 150 mM KCl, 0.5 mM $Mg(OAc)_2$, 1mM TCEP
CPF strep elution buffer	CPF wash buffer supplemented with 1.2 mg/ml desthiobiotin
CFIA lysis buffer	50 mM HEPES pH 7.9, 250 mM NaCl, 0.5 mM TCEP, 5% w/v glycerol, 2 μg/ml RNaseA (add fresh) and protease inhibitor tables (10 tablets per 120 ml of buffer) (Roche)
CFIA wash buffer	20 mM HEPES pH 7.9, 250 mM NaCl, 0.5 mM TCEP
CFIA strep elution buffer	CFIA wash buffer supplemented with 1.2 mg/ml desthiobiotin
Pulldown lysis buffer	100 mM HEPES pH 8, 300 mM NaCl, 5% glycerol, 1 mM TCEP
Pulldown wash buffer	100 mM HEPES pH 8, 300 mM NaCl, 1mM TCEP
Pulldown elution buffer	100 mM HEPES pH 8, 300 mM NaCl, 1mM TCEP, 6 mM desthiobiotin
Buffer A (Yth1)	50 mM HEPES pH 7.4, 150 mM NaCl
Buffer B (Fip1)	50 mM HEPES pH 7.4, 500 mM NaCl
Pap1 lysis buffer	50 mM HEPES pH 8.0, 1 M NaCl, 20 mM imidazole, 5% w/v glycerol, 1 mM TCEP, 2 μg/ml Dnase I, 2 μg/ml Rnase A and protease inhibitor mixture (Roche)
Pap1 buffer	50 mM HEPES pH 8.0, 1 M NaCl, 0.5 mM TCEP
Pap1 SEC buffer	50 mM HEPES pH 8.0, 150 mM NaCl, 1 mM TCEP
